# Construction of recombinant Marek’s disease virus co-expressing σB and σC of avian reoviruses

**DOI:** 10.3389/fvets.2024.1461116

**Published:** 2024-09-05

**Authors:** Li Gao, Li Zhong, Yongzhen Liu, Changjun Liu, Yanping Zhang, Hongyu Cui, Xiaole Qi, Jiayong Zhang, Jia Xu, Suyan Wang, Yuntong Chen, Yulu Duan, Kai Li, Yulong Gao, Xiaomei Wang

**Affiliations:** ^1^Avian Immunosuppressive Diseases Division, State Key Laboratory for Animal Disease Control and Prevention, Harbin Veterinary Research Institute, Chinese Academy of Agricultural Sciences, Harbin, China; ^2^College of Animal & Veterinary Sciences, Southwest Minzu University, Chengdu, China; ^3^Heilongjiang Provincial Center for Animal Disease Prevention and Control, Harbin, China; ^4^Institute of Urban Agriculture, Chinese Academy of Agricultural Sciences, Chengdu, China; ^5^Chengdu National Agricultural Science and Technology Center, Chengdu, China; ^6^Jiangsu Co-innovation Center for Prevention and Control of Important Animal Infectious Disease and Zoonoses, Yangzhou University, Yangzhou, China

**Keywords:** Marek’s disease virus, avian reovirus, σB, σC, vaccine

## Abstract

Avian reoviruses (ARVs) cause viral arthritis or tenosynovitis, resulting in poor weight gain and increased feed conversion ratios in chickens. In this study, we generated three Marek’s disease virus (MDV) recombinants, namely, rMDV-ARV-σB, rMDV-ARV-σC, and rMDV-ARV-σB + C, expressing ARV σB, σC, and both σB and σC, respectively. In rMDV-ARV-σB and rMDV-ARV-σC, the σB or σC gene was inserted into the US2 gene of MDV vaccine strain 814 using a fosmid-based rescue system. In rMDV-ARV-σB + C, the σB and σC genes were cloned into different expression cassettes, which were co-inserted into the US2 gene of the MDV 814 strain. In infected chicken embryo fibroblasts (CEFs), the recombinant virus rMDV-ARV-σB expressed σB, rMDV-ARV-σC expressed σC, and the rMDV-ARV-σB + C virus simultaneously expressed σB and σC. These recombinant viruses exhibited growth kinetics in CEFs similar to those of the parent MDV, and the inserted genes were stably maintained and expressed in the recombinant MDVs after 20 passages in cell cultures. These recombinant MDVs expressing σB and σC will provide potential vaccines against ARV infection in chickens.

## 1 Introduction

In chickens, avian reovirus (ARV) infections cause viral arthritis, stunting syndrome, and tenosynovitis, resulting in considerable economic losses in the poultry industry worldwide (1). Although most birds infected with this virus are infected via the fecal-oral route, ARV infection via the respiratory tract and egg transmission have also been reported. Chicken susceptibility to ARV infection is age-dependent, with older birds being established to be more resistant to both infections and viral-induced lesions (2).

ARV is a member of the genus *Orthoreovirus* in the family *Reoviridae*, which includes viruses comprising segmented genomes consisting of 10 genome segments of double-stranded (ds) RNA. The ARV genome can be divided into three size classes, namely large (L1, L2, and L3), medium (M1, M2, and M3), and small (S1, S2, S3, and S4), and expresses at least 12 primary translation products, of which eight and four are structural and non-structural proteins, respectively (3, 4). The σB protein is a minor component of the outer capsid of ARV (5, 6) that can induce group-specific neutralizing antibodies (7). Notably, σC is the only viral protein present in soluble extracts of infected cells, and this protein has been identified as a major protein for inducing the production of neutralizing antibodies against ARV (3, 8). Given their powerful immunogenicity, the σB and σC proteins have become optimal candidates for the construction of novel ARV vaccines (9–12).

Marek’s disease virus (MDV) is a highly cell-associated herpesvirus that causes Marek’s disease (MD), a neoplastic and neuropathic disease in chickens (13). MDV has a large genome, several regions of which are non-essential for viral replication. Furthermore, MDV vaccines can be inoculated into 1 day-old field chicks with high titers of maternal antibodies to establish early immunity (14). MDV vaccines can also induce lifetime immunity in chickens following the administration of just a single vaccination. These features make MDV a highly promising viral vector for the development of recombinant vaccines against ARV infections. In this study, by inserting the σB and σC genes of ARV into the genome of an MDV vaccine strain individually or conjointly, we succeeded in constructing three recombinant MDVs expressing σB, σC, or both σB and σC, which were evaluated *in vitro* for antigen expression, replication, and stability.

## 2 Materials and methods

### 2.1 Viruses, cells, and antibodies

As the parental virus for producing recombinant MDVs, we used the MDV serotype 1 (MDV1) 814 vaccine strain (15). These MDVs were propagated in chicken embryo fibroblasts (CEFs) prepared from 10 day-old specific-pathogen-free (SPF) chicken embryos. The mouse anti-σB monoclonal antibodies (MAb) and the mouse anti-σC MAb were prepared in our laboratory.

### 2.2 Construction of fosmids with insertion of the ARV σB and σC genes

The ARV σB and σC genes were individually inserted into pCAGGS vectors under the control of the CAG promoter (CMV enhancer/chicken β-actin promoter), and the resultant σB or σC cassette was then used to replace the gus gene in a pENTR-gus vector (Invitrogen) to obtain the attL1 and attL2 arms. In our preliminary studies, we constructed five fosmid clones containing genomic sequences spanning the entire genome of MDV1 vaccine strain 814 (16). To simplify the insertion of foreign genes into the MDV genome, fosmid 814E was modified by inserting a dual selection marker encoding the kanamycin resistance gene (KanR) and ccdB gene flanked by attR1 and attR2 sequences into the US2 gene of MDV using a Counter-Selection BAC modification kit (Gene Bridges Gmbh, Heidelberg, Germany).

**Figure 1:**
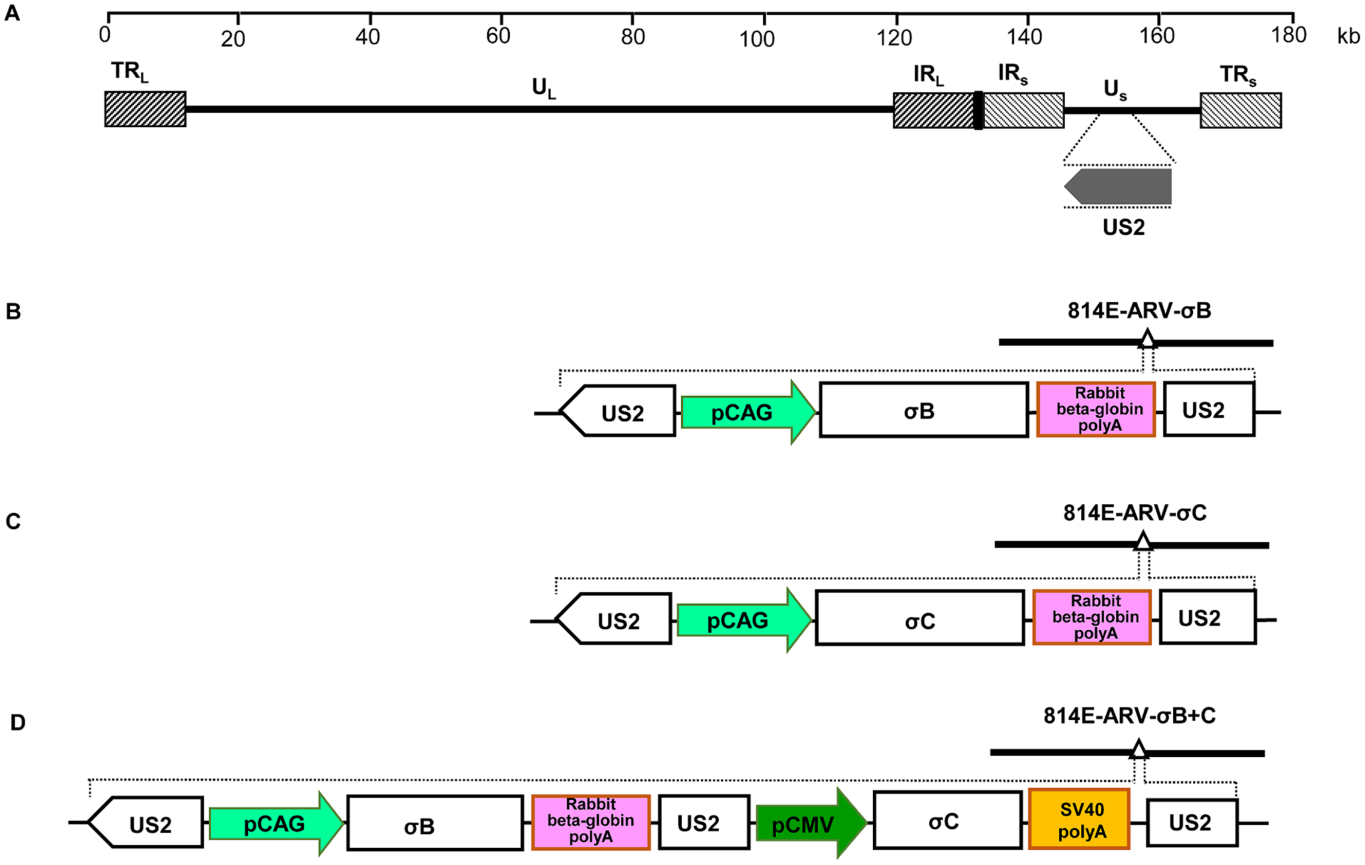
Construction of fosmids containing the ARV σB and σC genes. **(A)** The genomic structure of MDV vaccine strain 814. **(B)** Schematic diagram showing the recombinant fosmid 814E-ARV-σB containing the σB cassette inserted within the US2 gene in the MDV genome. **(C)** Schematic diagram showing the recombinant fosmid 814E-ARV-σC containing the σC cassette inserted within the US2 gene in the MDV genome. **(D)** Schematic diagram of the recombinant fosmid 814E-ARV-σB + C containing the σB and σC expression cassettes inserted within the US2 gene in the MDV genome.

To insert the σB or σC cassette into the MDV genome, entry plasmids were mixed with the modified fosmid 814E-Kan/ccdB, treated with LR Clonase II enzyme (Invitrogen), and then used to transform competent *Escherichia coli* EPI300-T1 cells. The resultant fosmids containing σB or σC cassette insertions were designated 814E-ARV-σB or 814E-ARV-σC, respectively (Figures 1B,C). To construct the recombinant fosmid 814E-ARV-σB + C co-expressing σB and σC, the σB gene was cloned into a pCAGGS vector under the control of the CAG promoter, and the σC gene was cloned into a pCI vector under control of the CMV promoter. Thereafter, the σB and σC cassettes were simultaneously cloned into a pENTR-gus vector and inserted into the US2 gene in fosmid 814E (Figure 1D).

### 2.3 Rescue of recombinant MDV from overlapping fosmid DNAs

For virus rescue, we used a set of five fosmids with or without σB and σC insertions. Viral DNA inserts were released from purified fosmids by digestion and the DNAs of each fosmid were used to transfect primary CEFs in 60 mm dishes using a Calcium Phosphate Transfection Kit (Invitrogen). Four days after transfection, the cells were trypsinized, seeded in 100 mm dishes, and monitored for cytopathic effects (CPE), with the CPE-positive samples being harvested and characterized by electron microscopy. To verify the correct insertion of the σB and σC genes into the MDV genome at the desired sites, the viral genomic DNA was analyzed by PCR and sequencing.

### 2.4 Confirmation of σB and σC expression

Expression of ARV σB and σC by recombinant MDVs was confirmed using an indirect immunofluorescence assay. Briefly, CEFs in six-well tissue culture plates were infected with the rescued viruses for 4 days. Thereafter, having aspirated the medium, the cells were fixed with absolute ethanol for 20 min at room temperature. The fixed cells were subsequently incubated with mouse anti-σB MAb or mouse anti-σC MAb for 60 min at 37°C, then reacted with FITC-conjugated goat anti-mouse IgG antibody (Sigma, St. Louis, MO) for 60 min at 37°C. After being washed five times, the cells were examined via fluorescence microscopy.

### 2.5 Growth properties and stability of the rescued viruses

To investigate the growth properties of the recombinant MDVs, cells cultured in six-well plates were inoculated with 100 plaque-forming units of the rescued viruses. The infected cells were harvested at different time points and serial dilutions were inoculated onto fresh CEFs. The plaques produced by the different dilutions were counted 5 days later. To evaluate the genetic stability of the recombinant MDVs, viruses were passaged 20 times in CEFs. Detection of the inserted σB and σC genes was carried out by PCR and sequencing. Expression of the σB and σC gene was confirmed by fluorescence assays as described above.

## 3 Results

### 3.1 Generation of recombinant MDVs containing the ARV σB and σC genes

**Figure 2:**
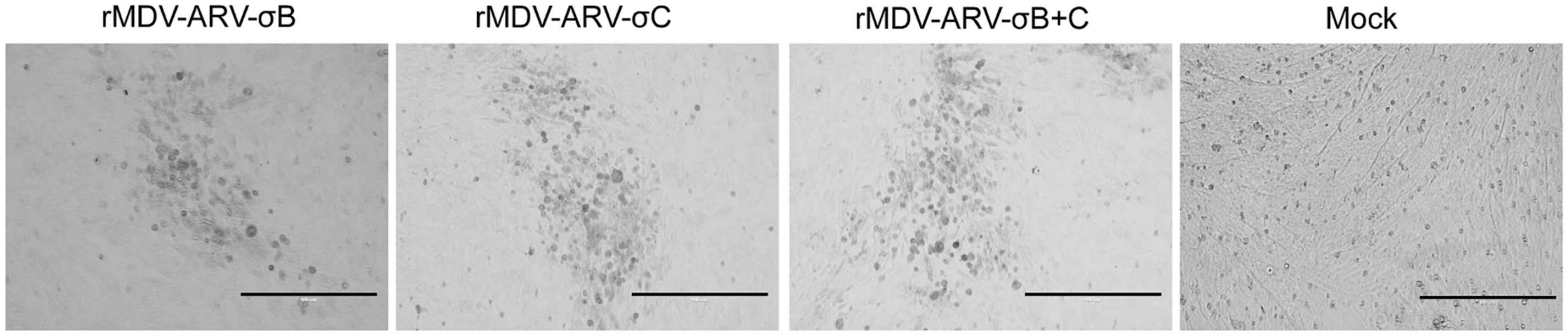
The cytopathic effects (CPE) induced by the recombinant MDVs containing σB and σC genes in chicken embryo fibroblasts (CEFs). CEFs were inoculated with the recombinant MDVs for 4 to 5 days prior to assessing CPE. Bar length, 200 μm.

For the construction of recombinant virus rMDV-ARV-σB and rMDV-ARV-σC, σB and σC cassettes were inserted into the US2 gene in the MDV genome, respectively, and the resultant recombinant fosmids (814E-ARV-σB or 814E-ARV-σC) were co-transfected with the parental fosmids into CEFs. For rMDV-ARV-σB + C, the σB and σC genes were cloned under the control of the CAG and CMV promoters, respectively, and the σB and σC cassettes were simultaneously inserted into the MDV genome. Having been blindly passaged in CEFs, MDV-typical plaques appeared in the CEFs transfected with the DNA combinations (Figure 2). Insertion of the σB and σC genes at the correct sites was confirmed by PCR and sequencing.

### 3.2 Expression of σB and σC from the recombinant MDVs

**Figure 3:**
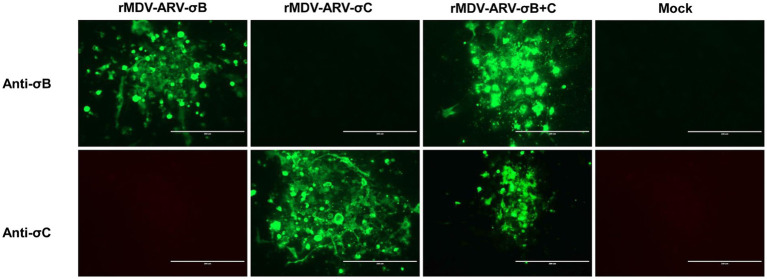
Detection of σB and σC expression by the recombinant viruses. Chicken embryo fibroblasts (CEFs) in six-well tissue culture plates were infected with the rescued viruses for 4 days, and the expression of σB and σC was determined using an indirect immunofluorescence assay with anti-σB and anti-σC antibodies. Bar length, 200 μm.

The expression of σB and σC by the recombinant viruses was confirmed via an indirect immunofluorescence assay. Cells infected with rMDV-ARV-σB or rMDV-ARV-σC reacted with anti-σB and anti-σC antibodies, respectively, whereas the cells infected with rMDV-ARV-σB + C reacted with both the anti-σB and anti-σC antibodies, emitting a green fluorescent signal (Figure 3). Contrastingly, we detected no reaction between the parental virus-infected cells and these antibodies. These results indicate that we had successfully generated recombinant MDVs expressing the ARV σB and σC genes.

### 3.3 Growth kinetics of the recombinant MDVs

**Figure 4:**
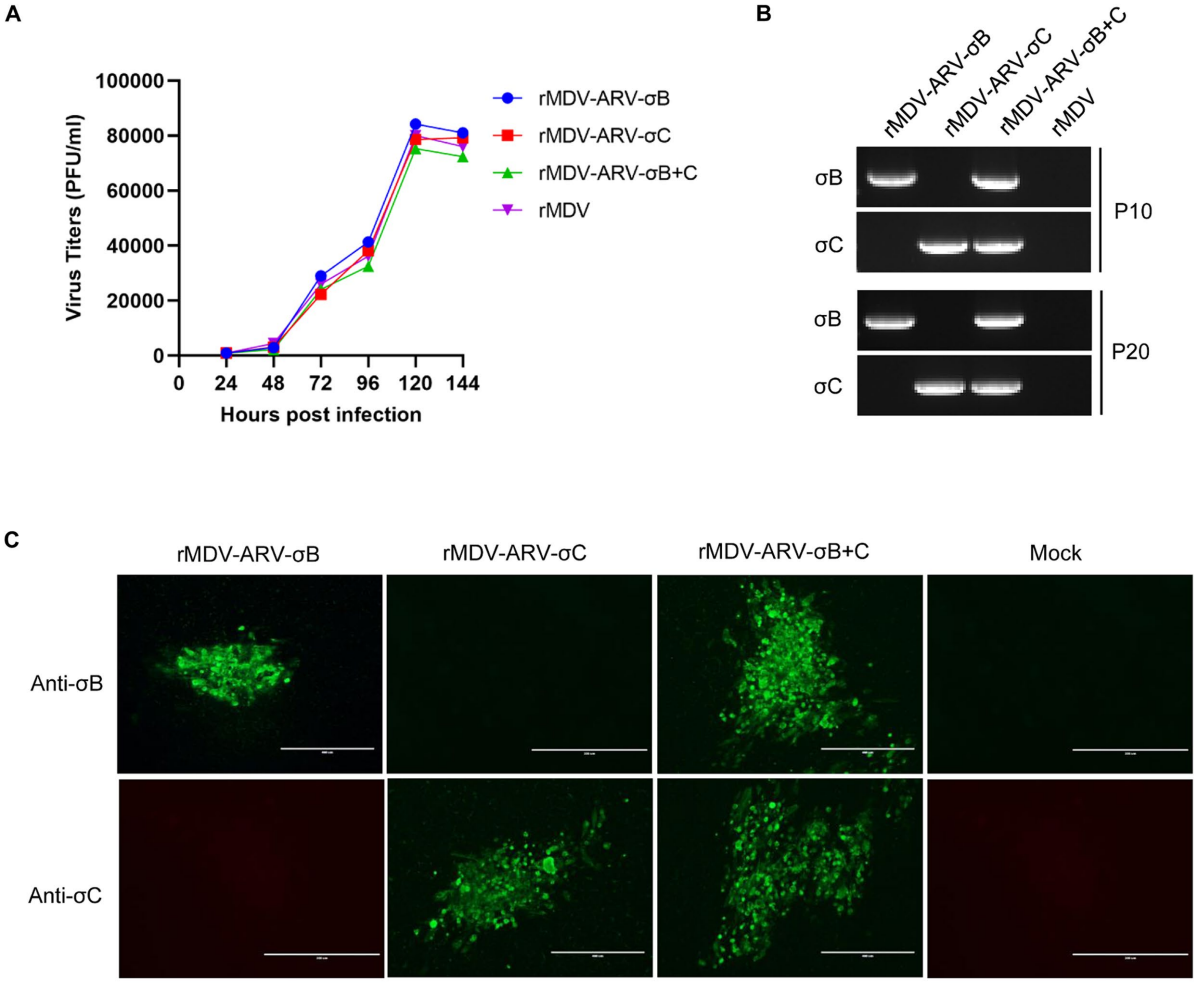
Growth kinetics and genetic stability of the recombinant MDVs in chicken embryo fibroblasts (CEFs). **(A)** Comparison of the replication kinetics of the recombinant MDVs and the parental virus (rMDV) in CEFs. **(B)** PCR detection of the σB and σC genes inserted in recombinant MDVs passaged 10 (P10) and 20 (P20) times in CEFs. **(C)** Confirmation of σB and σC expression by the recombinant MDVs passaged 20 times in CEFs using an immunofluorescence assay.

Replication of the recombinant viruses was analyzed and compared with that of parental viruses in CEFs. CEF cultures infected with the viruses were harvested at different time points for titration. The results showed that the growth kinetics and magnitude of the three recombinant viruses, rMDV-ARV-σB, rMDV-ARV-σC, and rMDV-ARV-σB + C were very similar to those of their parental viruses (Figure 4A). As shown in Figure 4A, the recombinant viruses and the parental virus achieved the highest replication level at 120 h post-infection with the viral titers of 10^4.92^, 10^4.87^, 10^4.89^, and 10^4.90^ PFU/mL (*p* > 0.05), indicating that insertion of the σB and σC genes in the US2 site had no significant effects on the replication of the MDV vaccine strains in CEFs.

### 3.4 Genetic stability of the recombinant MDVs

To investigate whether the inserted σB and σC genes can be stably maintained in the recombinant viruses, we passaged the viruses 20 times in CEFs. The σB and σC genes in both recombinants could be detected using PCR amplification (Figure 4B), and the inserted genes were correct as detected by sequencing. Furthermore, the σB and σC expression in recombinant virus-infected cells could still be detected by immunofluorescence after 20 passages (Figure 4C).

## 4 Discussion

In chicken farms, protection against ARV infections has historically been achieved via vaccination with commercial live and inactivated vaccines. The primary objectives of vaccination are to prevent the vertical transmission of ARV, provide maternal antibodies, and prevent clinical disease in progeny. In China, ARV infections have been successfully controlled in the past few years through the use of vaccines, although the current vaccines against ARV may not provide full protection and can cause adverse reactions (12). Since 2013, ARV infection has been increasingly detected in broilers in China (17), and the viral arthritis and severe immunosuppression caused by ARV variants pose a new threat to the broiler industry and breeding stocks. Additionally, infection with MDV is a perennial problem, and co-infection with ARV and MDV is a frequent annual occurrence in China and other countries in which these viruses are endemic. In this context, the development of a bivalent vaccine candidate that can protect against both these viral infections is of particular importance.

In previous studies, multiple types of genetically engineered vaccines have been generated to prevent ARV infections. For example, the DNA vaccines SL7207 (pVAX-σB), SL7207 (pVAX-σC), and SL7027 (pVAX-σB-σC) have, respectively, been demonstrated to confer 50, 75, and 87.5% protection against ARV infections in chickens (18). In a further study, the full-length (residues 1–326) and two partial fragments of σC (residues 122–326 and 192–326) were produced in *Escherichia coli*, among which, the 122–326 fragment was found to induce significantly higher levels of anti-ARV antibodies than the shorter fragment or the full-length σC (19). Furthermore, the coding sequence of the σC protein has been expressed in *Schizosaccharomyces pombe*, and a high dose of 250 μg purified yeast-expressed σC protein was found to provide 91% protection against ARV infection in chickens (12). Additionally, the σC gene has been cloned into the NDV genome and the resultant virus rNDV-R2B-σC was found to induce both humoral and cell-mediated immune responses in birds and conferred complete protection against virulent NDV and ARV challenges (20).

The ARV σB and σC proteins are the two main factors that can induce the production of neutralizing antibodies in chickens. In this study, we used an MDV vaccine strain as the vector to mediate the delivery of the σB and σC genes. As an avian herpesvirus, MDV has a large genome and is not susceptible to maternal antibodies owing to its cell-to-cell transmission properties. Additionally, given the persistent nature of MDV infection, MDV-vectored vaccines can contribute to inducing a long-term immune response (21, (22). In the present study, the σB and σC genes were independently inserted into the MDV genome to generate two recombinant MDVs, rMDV-ARV-σB and rMDV-ARV-σC, expressing σB and σC, respectively. Moreover, the σB and σC expression cassettes were conjointly inserted into the MDV genome, thereby yielding the recombinant virus rMDV-ARV-σB + C expressing both σB and σC genes. We previously inserted the VP2 gene of infectious bursal disease virus into different sites of the MDV genome, and the recombinant virus r814US2VP2 with VP2 insertion in the US2 site conferred the highest protection level compared to those inserted with VP2 gene in other sites (23); we therefore chose the US2 site in this study for the insertion of ARV σB and σC genes. Our findings indicated that the inserted σB and σC genes were stably maintained in the US2 site and expressed in the infected cells, and that insertion of the σB and σC genes in the MDV genome had no significant detrimental effects concerning the replicative capacity of the parental virus. We believe that these recombinant viruses expressing σB and σC could have significant potential applications as MDV-vectored vaccines for combatting ARV infections in chickens.

## Data availability statement

The original contributions presented in the study are included in the article/Supplementary material, further inquiries can be directed to the corresponding authors.

## Author contributions

LG: Methodology, Investigation, Validation, Writing – original draft. LZ: Conceptualization, Investigation, Methodology, Validation, Writing – original draft. YL: Conceptualization, Formal analysis, Methodology, Resources, Writing – review & editing. CL: Methodology, Resources, Writing – review & editing. YZ: Methodology, Resources, Writing – review & editing. HC: Methodology, Resources, Writing – review & editing. XQ: Methodology, Resources, Writing – review & editing. JZ: Methodology, Resources, Writing – review & editing. JX: Methodology, Resources, Writing – review & editing. SW: Methodology, Resources, Writing – review & editing. YC: Methodology, Resources, Writing – review & editing. YD: Methodology, Resources, Writing – review & editing. KL: Conceptualization, Funding acquisition, Methodology, Validation, Writing – original draft. YG: Conceptualization, Supervision, Validation, Writing – review & editing. XW: Conceptualization, Supervision, Validation, Writing – review & editing.

## Supplementary material

SUPPLEMENTARY FIGURE S1 Detection of the σB and σC genes inserted in the recombinant MDVs passaged 10 (P10) times in CEFs by PCR.

SUPPLEMENTARY FIGURE S2 Detection of the σB and σC genes inserted in the recombinant MDVs passaged 20 (P20) times in CEFs by PCR.
