# Supplementary figures and images for: Construction of recombinant Marek’s disease virus co-expressing σB and σC of avian reoviruses
Source: Front Vet Sci. 2024 Sep 5;11:1461116. doi: 10.3389/fvets.2024.1461116 (PMC11410777; doi:10.3389/fvets.2024.1461116)

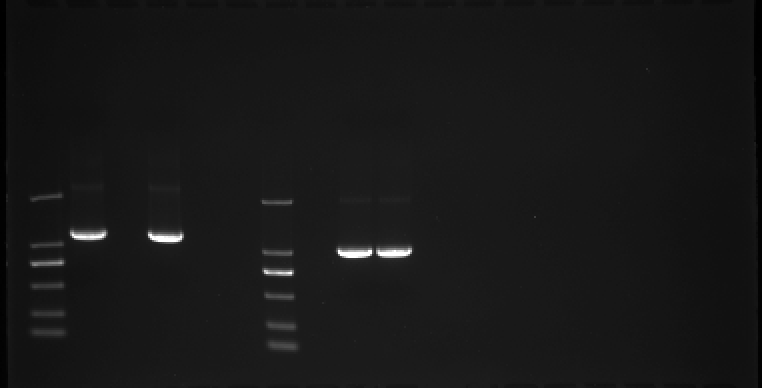

Supplement: SUPPLEMENTARY FIGURE S1 — Detection of the σB and σC genes inserted in the recombinant MDVs passaged 10 (P10) times in CEFs by PCR. [file Image_1.png]

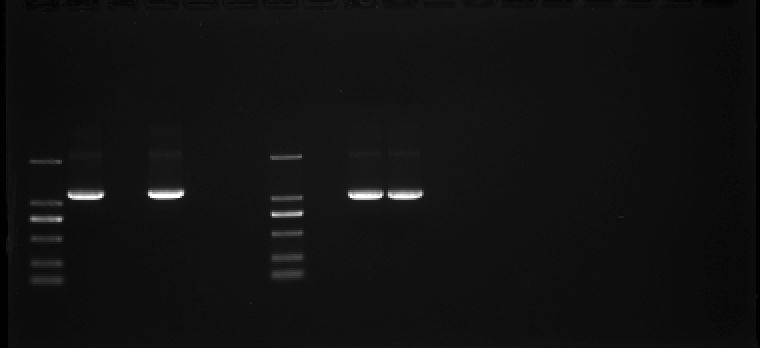

Supplement: SUPPLEMENTARY FIGURE S2 — Detection of the σB and σC genes inserted in the recombinant MDVs passaged 20 (P20) times in CEFs by PCR. [file Image_2.png]
